# Integrated transcriptomic profiling implicates MAPK14/p38α as a candidate shared molecular target at the sepsis–postoperative delirium interface with computational evidence for a losartan–MAPK14 association

**DOI:** 10.1371/journal.pone.0357109

**Published:** 2026-08-26

**Authors:** Wei-Jia Kong, Jing-Xian Xue, Lei Yu, Qing-Shan Zhou, Huai-Qi Zhang, Jun Jin, Jiang-Tao Deng

**Affiliations:** 1 Department of Critical Care Medicine, The University of Hong Kong-Shenzhen Hospital, Shenzhen, Guangdong, China; 2 Department of Pain Medicine, The Eighth Affiliated Hospital, Sun Yat-sen University, Shenzhen, Guangdong, China; INNN: Instituto Nacional de Neurologia y Neurocirugia Manuel Velasco Suarez, MEXICO

## Abstract

**Background:**

Sepsis and postoperative delirium (POD) may share inflammatory and neuroimmune mechanisms, but their common molecular basis remains unclear. This study aimed to identify candidate shared molecular targets and explore a clinically feasible pharmacological candidate linked to the prioritized target.

**Methods:**

Multi-cohort transcriptomic data from the Gene Expression Omnibus were integrated. Marked test-statistic inflation in the sepsis discovery cohort GSE65682 was addressed using a Removal of Unwanted Variation 2 (RUV2)-style adjustment based on the 10% lowest-variance genes and four estimated unwanted factors. Transcriptome-wide directional concordance between sepsis and POD was assessed using a rank-rank hypergeometric overlap-like analysis. Separately, RUV2-corrected sepsis differentially expressed genes were intersected with nominally significant POD candidate genes, and directionally concordant genes underwent functional-enrichment and protein–protein interaction analyses. Candidate genes retained from the protein–protein interaction (PPI) network analysis were further evaluated through cross-cohort expression analysis and SHAP-based feature importance analysis. Following target prioritization, target-guided pharmacological assessment, molecular docking, and immune-cell signature analysis were performed.

**Results:**

RUV2-style adjustment reduced the genomic inflation factor in GSE65682 from 41.49 to 6.09, although residual inflation remained. The corrected analysis identified 440 sepsis-associated differentially expressed genes. Forty-four genes overlapped with the exploratory POD candidate set, of which 22 showed concordant directions of change. PPI analysis identified seven network-connected candidates. MAPK14 showed consistent disease-associated upregulation across the sepsis discovery cohort, an independent sepsis cohort, and the POD cohort, and had the highest SHAP contribution. Following prioritization of MAPK14, pharmacological candidates linked to this target were evaluated, leading to the selection of losartan as a clinically feasible candidate for exploratory assessment. Molecular docking suggested structural plausibility but did not establish direct binding or functional inhibition. MAPK14 expression was also positively associated with a neutrophil-predominant immune signature.

**Conclusions:**

These findings implicate MAPK14/p38α as a candidate shared molecular target between sepsis and POD. The losartan–MAPK14 association remains computational and hypothesis-generating and requires experimental and prospective clinical validation.

## Introduction

Sepsis is a life-threatening organ dysfunction syndrome caused by a dysregulated host response to infection [1], remaining a leading cause of morbidity and mortality in intensive care units (ICUs) worldwide [2]—affecting an estimated 48.9 million individuals annually and accounting for approximately 11 million deaths, representing nearly 20% of all-cause global mortality [3]. The deleterious effects of sepsis extend beyond peripheral organs, frequently compromising the central nervous system (CNS) and manifesting as acute brain dysfunction [4]. In perioperative and critical care settings, postoperative delirium (POD) is one of the most prevalent and severe neurocognitive complications, with reported incidences of 10–60% depending on patient age, surgical type, and diagnostic criteria applied [5]. The co-occurrence of sepsis and POD imposes a profound synergistic burden [6]; delirium occurring in critically ill patients—including those with sepsis and those in the perioperative setting—is independently associated with prolonged mechanical ventilation, increased ICU length of stay [7], accelerated long-term cognitive decline [8], and elevated mortality [9,10]. Despite the implementation of source control and organ support, specific pharmacological interventions for this acute neurocognitive complication remain notably scarce, primarily due to an incomplete understanding of its underlying pathogenesis.

While the exact molecular mechanisms linking systemic sepsis to the onset of POD remain elusive, prevailing hypotheses suggest the involvement of profound neuroinflammation, blood-brain barrier (BBB) disruption, and cerebral microcirculatory failure [11]. Furthermore, transcriptomic datasets from patients with both sepsis and POD remain scarce, which limits direct investigation of the molecular links between systemic inflammation and acute neurocognitive dysfunction. Therefore, comparing sepsis and POD as two related but independently profiled conditions provides a practical approach to identify shared molecular signals. Differentially expressed genes (DEGs) that overlap between the two conditions and show the same direction of change are unlikely to represent random overlap alone; rather, they may reflect common immune-inflammatory processes involved in both conditions. Identifying these shared processes may help explain how systemic inflammation creates a biological environment that increases the risk of delirium in vulnerable patients.

Because POD currently lacks broadly accepted mechanism-based pharmacological prevention or treatment strategies, shared molecular hubs may also provide a rational starting point for drug-repurposing exploration. Therefore, this study aimed to systematically characterize the shared transcriptomic features of sepsis and POD, identify directionally consistent shared DEGs, prioritize hub genes through protein-protein interaction (PPI) network analysis and machine-learning-based feature interpretation, and explore whether the prioritized molecular hub was linked to clinically relevant candidate compounds. Through this stepwise framework, we sought to generate mechanistic hypotheses for sepsis-associated POD and to evaluate the potential relevance of losartan only after identification of the shared hub-gene signal.

## Materials and methods

### Data acquisition

The transcriptomic profiles analyzed in this research were retrieved from the Gene Expression Omnibus (GEO) repository at the National Center for Biotechnology Information (NCBI). The roles, platforms, sample sources, sample sizes, and available demographic and clinical characteristics of the included datasets are summarized in Table 1.

**Table 1:** Characteristics of the analyzed transcriptomic datasets.

Characteristic	GSE65682	GSE163943	GSE95233
**Role in the study**	Sepsis discovery cohort	POD cohort	Independent sepsis validation cohort
**Phenotype**	Sepsis	Postoperative delirium	Septic shock
**Organism**	Homo sapiens	Homo sapiens	Homo sapiens
**Tissue/ sample source**	Whole blood leukocytes	Peripheral blood	Whole blood
**Platform**	GPL13667	GPL26963	GPL570
**Total samples analyzed (n)**	802	8	79
**Disease samples (n)**	760	4	68
**Control samples (n)**	42	4	11
**Country/ region**	Netherlands/ United Kingdom; per-sample country not provided in GEO	China	France
**Age**	17-93 years (median 63)	Not reported in public metadata	25-85 years (median 66)
**Sex**	Male 470; female 332	Not reported in public metadata	Male 50; female 29
**Comorbidities/ clinical metadata reported in public source**	Partial clinical metadata available, including diabetes mellitus, pneumonia diagnosis, thrombocytopenia, and ICU-acquired infection; comprehensive comorbidity profile not reported in public metadata	Not reported in public metadata	Not reported in public metadata
**Genes analyzed (n)**	11762	44824	22878

GSE65682 (GPL13667, Affymetrix Human Gene 1.1 ST Array) served as the sepsis discovery cohort; GSE95233 (GPL570, Affymetrix Human Genome U133 Plus 2.0 Array) was used as an independent sepsis validation cohort; GSE163943 (GPL26963, Agilent-079487 Arraystar Human long non-coding RNA (lncRNA) microarray V3) was employed for POD analysis. Disease, number of patients in the disease group; Control, number of subjects in the control group. Abbreviations: GEO, Gene Expression Omnibus; GSE, Gene Expression Omnibus Series; GPL, GEO platform; POD, postoperative delirium; ICU, intensive care unit; lncRNA, long non-coding RNA.

### Ethics statement

Ethics approval and informed consent were not required for this study because all analyses were performed using publicly available, de-identified datasets and open-access databases. No new human participants, human specimens, animal experiments, or field research were involved.

### Data preprocessing and gene annotation

Processed GEO series-matrix expression data and corresponding platform annotation files were retrieved for each dataset. Expression ranges were inspected, and log2 transformation was applied when required based on the distribution of expression values. Probe identifiers were mapped to gene symbols using the corresponding platform annotation files. Probes without valid gene-symbol annotation were removed, and multiple probes mapping to the same gene were collapsed to gene-level expression values using limma::avereps(). Sample group labels were assigned and verified according to the GEO metadata. Because processed GEO series-matrix files rather than raw array files were used, raw array-level quality-control procedures could not be repeated uniformly across datasets; therefore, quality control was performed at the expression-matrix level.

### Differential expression analysis

Differential-expression statistics were initially estimated separately for each dataset using the limma package with empirical Bayes moderation. Benjamini–Hochberg-adjusted P values were calculated for all genes. The initial GSE65682 and GSE163943 results were used to characterize overall transcriptomic patterns, perform the RRHO-like analysis, and assess test-statistic behavior. For GSE163943, no genes remained significant after FDR correction because of the limited sample size; therefore, a nominal threshold of P < 0.05 and |log2FC| > 0.263 was used to define exploratory POD candidate genes for visualization and shared-signal generation. Test-statistic behavior was evaluated using quantile–quantile plots and genomic inflation factors. Volcano plots and heatmaps were generated using ggplot2 and pheatmap. The final GSE65682 DEG set was defined after Removal of Unwanted Variation 2 (RUV2)-style adjustment, as described below.

### Transcriptome-wide concordance analysis

Transcriptome-wide directional concordance between sepsis and POD was assessed using a binned rank-rank hypergeometric overlap-like (RRHO-like) analysis. Genes in GSE65682 and GSE163943 were ranked according to signed differential-expression statistics, and concordant and discordant enrichment across the two ranked gene lists was evaluated using hypergeometric tests. Multiple-testing correction across the RRHO-like grid was performed using the Benjamini–Hochberg method. Because genome-wide ranked gene lists were used, this analysis did not rely on predefined DEG significance cutoffs.

### RUV2-style adjustment and identification of shared candidate genes

To address test-statistic inflation in GSE65682, an RUV2-style adjustment was implemented using low-variance genes as empirical control genes [12,13]. Candidate control sets comprising the lowest-variance 5%, 10%, and 20% of genes were evaluated across k values from 1 to 10. For each parameter combination, the specified number of unwanted-variation factors was estimated from the corresponding empirical control-gene set and included as covariates in the limma design matrix. The final specification was selected by jointly considering the genomic inflation factor and the stability of the resulting DEG count.

### Functional Enrichment and Protein–Protein Interaction Network Analysis

Directionally concordant shared candidate genes were subjected to Gene Ontology enrichment analysis using the clusterProfiler package, with P values adjusted using the Benjamini–Hochberg method. Protein–protein interaction data were obtained from STRING and analyzed using igraph. Four network-topological metrics—Degree, maximal clique centrality, eigenvector centrality, and maximum neighborhood component—were calculated, and genes with non-zero scores across all four metrics were retained as network-connected candidate genes.

### Machine Learning and SHAP-Based Feature Importance

An extreme gradient boosting (XGBoost) classifier was trained using expression profiles of the hub genes in the sepsis discovery cohort to assess relative feature contribution. The POD cohort was not used for model training because of its limited sample size. SHapley Additive exPlanations (SHAP) values were calculated using shapviz, and genes were ranked according to mean absolute SHAP values. To assess potential overfitting, repeated stratified 5-fold cross-validation with 20 repeats was additionally performed, and model calibration was evaluated using the Brier score and calibration curve analysis.

### Cross-cohort expression validation and ROC Analysis

Hub-gene expression levels were compared between disease and control groups across cohorts using Wilcoxon rank-sum tests. Single-gene receiver operating characteristic (ROC) analyses were performed as exploratory assessments of individual hub-gene discrimination, and bootstrap 95% confidence intervals (CIs) were calculated for area under the ROC curve (AUC) estimates. ROC results from the POD cohort were interpreted cautiously because of the 4 vs 4 sample size.

### Target-guided pharmacological candidate assessment

After prioritization of the candidate shared molecular target, associated pharmacological candidates were considered based on available drug–gene evidence, prior literature, clinical availability, established human use, and mechanistic relevance. The selected candidate compound was subsequently evaluated using the Comparative Toxicogenomics Database, ChEMBL, and SwissTargetPrediction. Human-associated targets were harmonized across the three resources, and their overlap with the network-connected candidate genes was examined.

### Molecular docking analysis

The selected pharmacological candidate and prioritized molecular target were subjected to molecular docking. The receptor structure was obtained from the Protein Data Bank (PDB ID: 1A9U), and the ligand structure was retrieved from PubChem (CID: 3961). The receptor and ligand were prepared and converted to PDBQT format. Docking was performed using AutoDock Vina v1.2.7, with the search grid centered on the ATP-binding pocket and the exhaustiveness parameter set to 32. Predicted poses were retained and ranked according to their Vina docking scores. The top-ranked pose was used for structural visualization, and minimum heavy-atom distances between the ligand and selected pocket residues were calculated across the predicted poses.

### Immune-cell signature analysis

Immune-cell-associated transcriptional signature scores were calculated from the normalized gene-expression matrices using single-sample Gene Set Enrichment Analysis. Differences in signature scores between disease and control groups were assessed using the Wilcoxon rank-sum test. Correlations between the expression of the prioritized candidate gene and immune-cell signature scores were evaluated across cohorts. P values from multiple immune-cell comparisons were adjusted using the Benjamini–Hochberg method.

## Results

### Dataset characteristics, differential expression profiles, and cross-condition transcriptomic concordance

**Fig 1:**
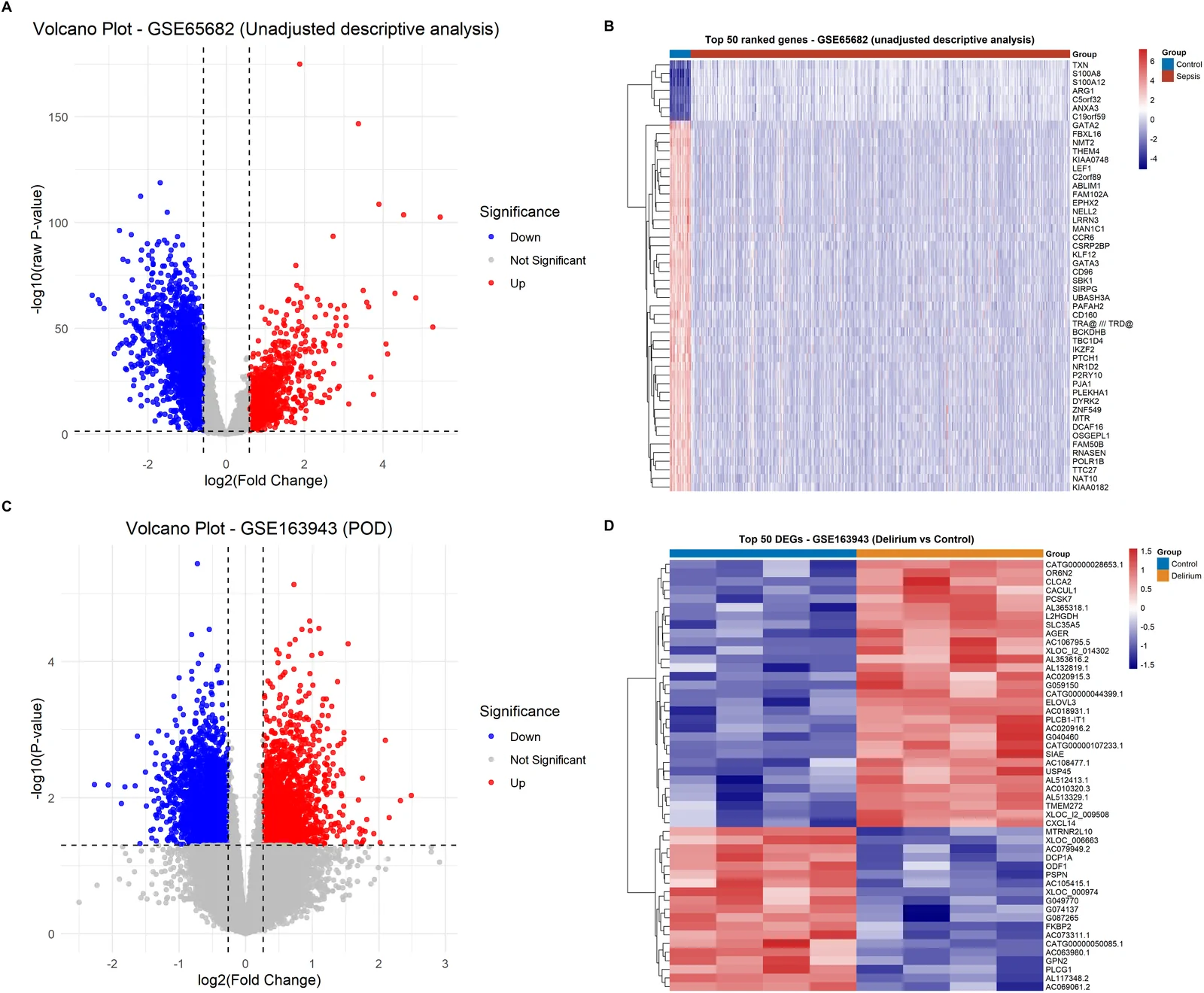
Transcriptomic profiling of differentially expressed genes in sepsis and postoperative delirium. (A) Volcano plot of differentially expressed genes (DEGs) in the sepsis discovery cohort GSE65682. Red indicates upregulated genes and blue indicates downregulated genes, based on FDR < 0.05 and |log_2_FC| > 0.585. (B) Heatmap of the top 50 genes in GSE65682 ranked by nominal P value; rows were Z-score normalized and columns were ordered by group. (C) Volcano plot of DEGs in the POD cohort GSE163943, based on the nominal threshold of P < 0.05 and |log_2_FC| > 0.263. These POD DEGs were used for exploratory visualization because no gene survived FDR correction in this underpowered cohort. (D) Heatmap of the top 50 genes in GSE163943 ranked by nominal P value. DEG, differentially expressed gene; POD, postoperative delirium; FDR, false discovery rate; FC, fold change. GSE, Gene Expression Omnibus Series.

Two human cohorts were incorporated in this step. In the well-powered sepsis discovery cohort (GSE65682, n = 802), differential expression analysis identified 1,005 upregulated and 2,154 downregulated genes, yielding 3,159 DEGs at Benjamini–Hochberg-adjusted P < 0.05 and |logFC| > 0.585 (Fig 1A–B). In the POD cohort (GSE163943, n = 8), differential expression analysis identified 1,889 upregulated and 2,465 downregulated genes, yielding 4,354 nominal DEGs at P < 0.05 and |logFC| > 0.263 (Fig 1C–D). Because this cohort was severely underpowered, the POD DEGs were called at a nominal significance level and are interpreted as exploratory; notably, no genes survived FDR correction in this cohort. To ensure that the cross-condition shared signal did not depend on this threshold-based DEG definition, convergence between sepsis and POD was additionally established using a threshold-independent, rank-based RRHO-like concordance analysis. Volcano plots illustrated the global distribution of dysregulated genes, while heatmaps of the top-ranked DEGs showed distinct expression patterns between disease and control samples in both cohorts (Fig 1).

### Statistical power and transcriptome-wide concordance

**Fig 2:**
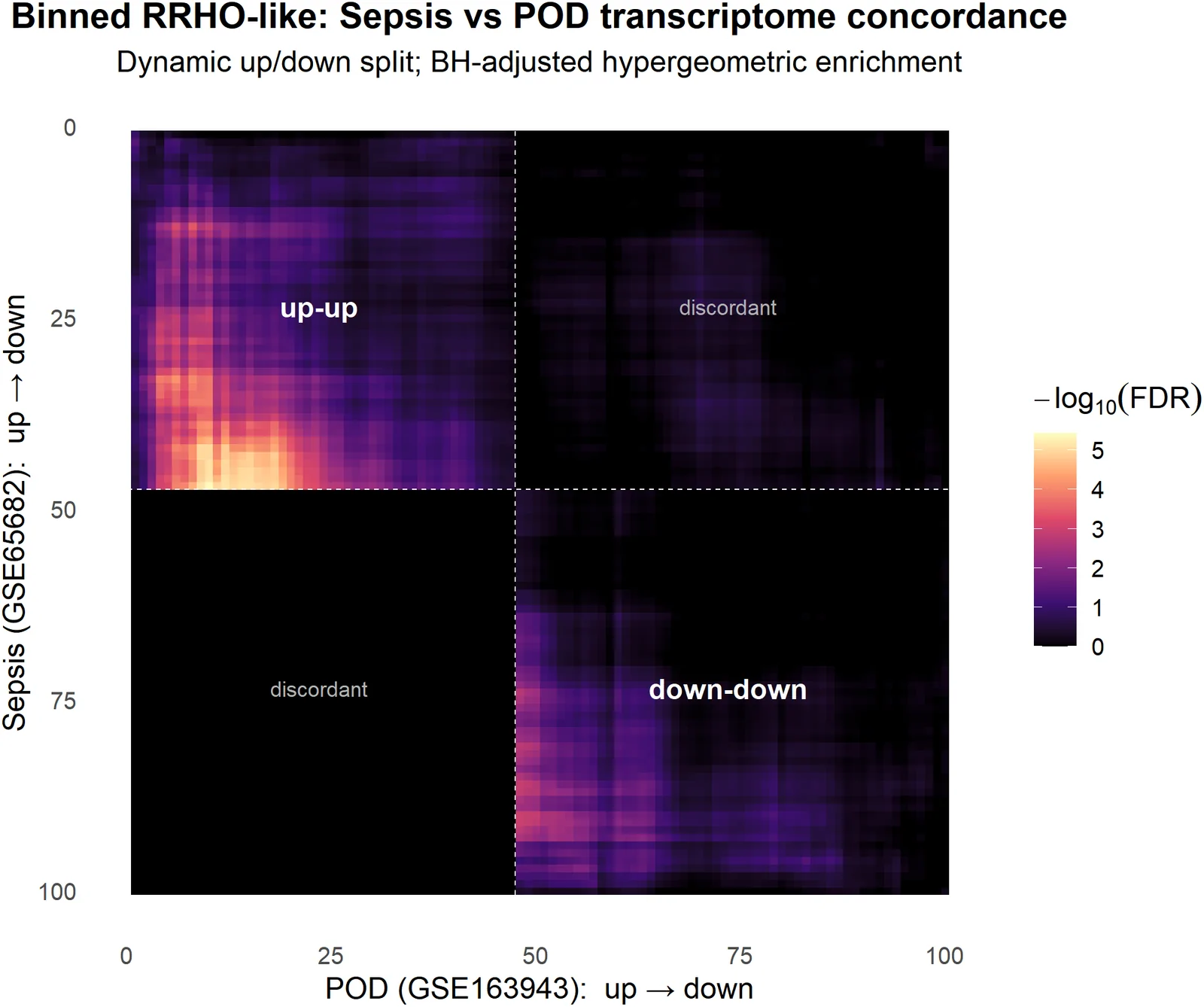
Threshold-independent binned RRHO-like analysis reveals concordant transcriptomic dysregulation between sepsis and POD. RRHO-like analysis was performed using genome-wide ranked differential-expression statistics from GSE65682 (sepsis) and GSE163943 (POD). Genes were ranked from upregulated to downregulated within each dataset, and enrichment was assessed across binned rank thresholds using hypergeometric tests with Benjamini–Hochberg adjustment. The color scale represents −log10(FDR), with brighter colors indicating stronger enrichment. Concordant enrichment was observed in the up–up and down–down quadrants, with the strongest signal in the up–up quadrant (peak −log10[FDR] = 5.4) and a weaker but evident signal in the down–down quadrant (peak −log10[FDR] = 3.0). Discordant quadrants showed minimal enrichment (maximum −log10[FDR] = 0.8), supporting transcriptome-wide directional concordance between sepsis and POD. RRHO-like, rank–rank hypergeometric overlap-like; POD, postoperative delirium; FDR, false discovery rate. GSE, Gene Expression Omnibus Series.

Because the POD cohort was severely underpowered and no suitable additional POD GEO cohort was available to increase the sample size, no genes survived FDR correction in GSE163943. Formal power analysis further confirmed the limited ability of this cohort to detect genome-wide differential-expression signals (Supplementary S2 Table). Therefore, nominally significant genes in the POD cohort were treated as exploratory candidate signals and used for visualization and shared-signal generation, rather than as FDR-confirmed POD DEGs. We next performed a threshold-independent RRHO-like analysis using genome-wide ranked differential-expression statistics from both datasets (Fig 2). This analysis did not rely on the nominal POD DEG list, but instead tested whether genes with stronger evidence of differential expression showed concordant directional enrichment across sepsis and POD. Significant concordant enrichment was observed, with the strongest signal in the up–up quadrant (peak −log10[FDR] = 5.4) and a weaker signal in the down–down quadrant (peak −log10[FDR] = 3.0), whereas discordant quadrants showed minimal enrichment (maximum −log10[FDR] = 0.8). Directional concordance among genes nominally significant in both datasets was also significant (517 of 921 genes, 56.1%; Fisher’s exact test: OR = 1.64, P = 3.2 × 10 − 4). Together, these findings support a shared sepsis–POD transcriptomic signal driven, at least in part, by concordant differential-expression patterns, rather than being solely attributable to the relaxed nominal significance threshold used for the POD cohort.

### RUV2-style adjustment and functional characterization of shared candidate genes

The initial unadjusted differential-expression model showed marked genomic inflation (λGC = 41.49; Supplementary S1 Table), indicating that unmeasured unwanted variation was not adequately accounted for. Because predefined control probes and complete technical batch information were unavailable, an RUV2-style correction was applied using low-variance genes as empirical negative controls to estimate unwanted factors. Candidate combinations of the proportions of empirical controls and the numbers of unwanted factors were evaluated to identify a parsimonious model that reduced genomic inflation while maintaining relatively stable DEG counts. Based on the observed stabilization of both metrics, the 10% of genes with the lowest variance and k = 4 unwanted factors were selected for the final sepsis model (S1 Fig). After correction, λGC decreased to 6.09, indicating a substantial, although incomplete, reduction in genomic inflation (S2 and S4 Figs). The corresponding QQ plots and λGC estimates for the independent sepsis validation cohort GSE95233 and the POD cohort GSE163943 are presented in S3 and S5 Figs, respectively.

**Fig 3:**
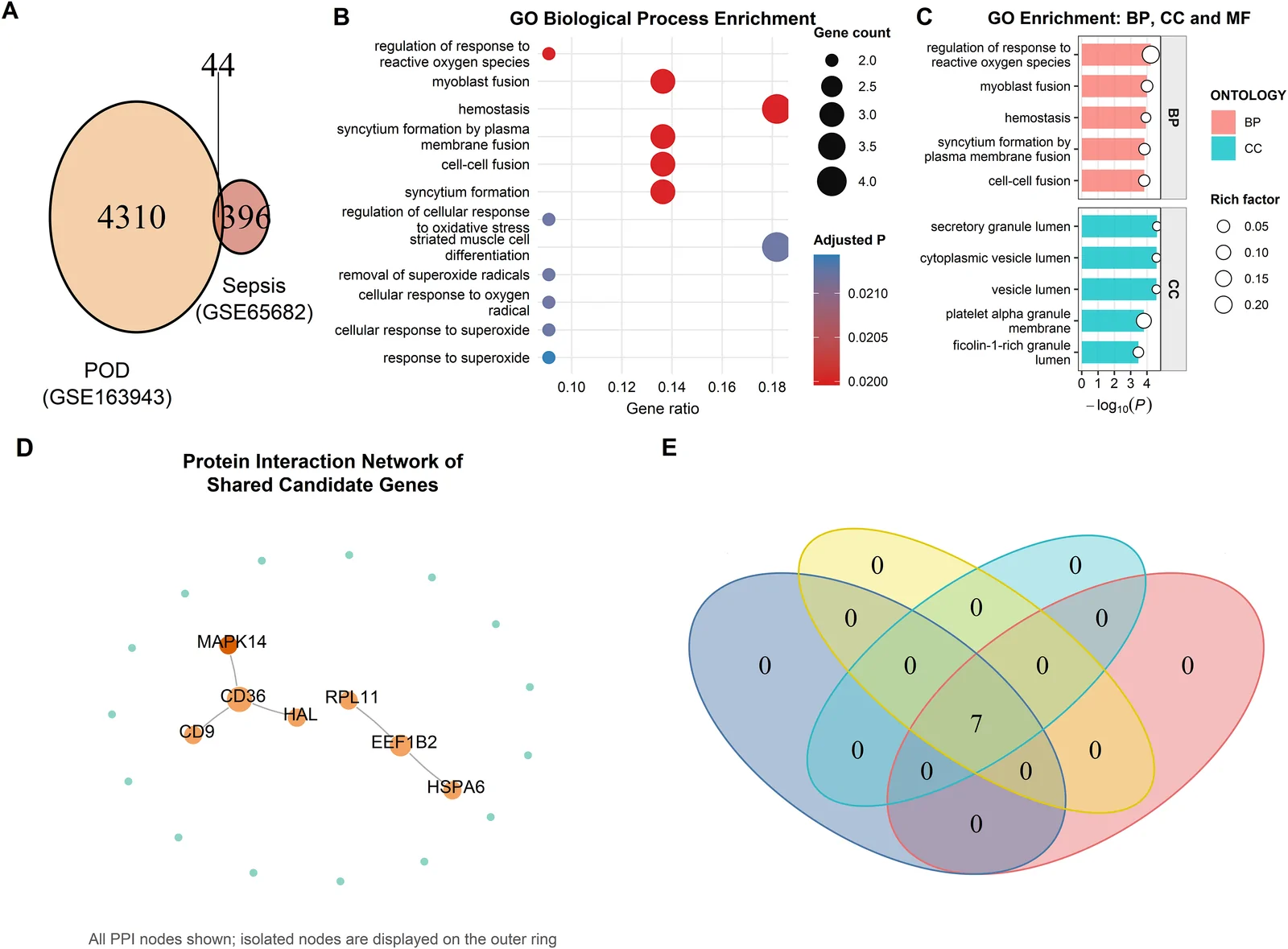
Functional characterization of candidate shared differentially expressed genes between sepsis and postoperative delirium. (A) Venn diagram showing the overlap between RUV2-corrected sepsis DEGs (n = 440) and nominally defined POD candidate DEGs (n = 4,354); 22 of the 44 overlapping genes showed directionally concordant changes. (B) GO-BP enrichment of the 22 directionally concordant candidate shared DEGs. (C) GO enrichment across BP and CC categories. (D) STRING PPI network comprising 22 nodes and 5 unique edges; node size represents Degree, and MAPK14 is highlighted. (E) Overlap of genes with non-zero MCC, Degree, eigenvector centrality, and MNC scores; seven genes were shared across all four metrics. DEG, differentially expressed gene; POD, postoperative delirium; GO, Gene Ontology; BP, biological process; CC, cellular component; STRING, Search Tool for the Retrieval of Interacting Genes/Proteins; PPI, protein–protein interaction; MCC, maximal clique centrality; MNC, maximum neighborhood component.

Using the RUV2-corrected sepsis DEG set, Venn diagram analysis identified 44 genes overlapping with the nominally defined POD candidate DEG set. Among these genes, 22 showed directionally consistent changes, including 9 genes upregulated and 13 genes downregulated in both conditions (Fig 3A). Gene Ontology enrichment analysis revealed a heterogeneous functional profile among these directionally consistent candidate genes. Enriched biological processes primarily involved regulation of responses to reactive oxygen species, hemostasis, and cell-fusion or differentiation-related processes, whereas enriched cellular component terms were dominated by secretory and vesicular lumens, including secretory granule, ficolin-1-rich granule, and specific granule compartments, together with platelet alpha-granule and sarcomeric structures (Fig 3B,C). These findings suggest that the directionally consistent shared-gene set reflects a mixed stress-response, granule-associated, hemostatic, and tissue-remodeling signature rather than a single dominant canonical immune pathway.

### PPI Network Analysis and Hub Gene Identification

The 22 directionally consistent candidate shared DEGs were mapped to the Search Tool for the Retrieval of Interacting Genes/Proteins (STRING) database, and all 22 genes were successfully annotated. The resulting PPI network comprised 22 nodes and 5 unique edges. Four network-topological metrics—maximal clique centrality (MCC), Degree, eigenvector centrality, and maximum neighborhood component (MNC)—were calculated for all nodes. Seven genes—CD36, EEF1B2, MAPK14, HAL, HSPA6, RPL11, and CD9—showed non-zero scores across all four metrics and were retained as network-connected candidate shared genes. CD36 and EEF1B2 had the highest Degree values, at 3 and 2, respectively, while the remaining five genes each had a Degree value of 1 (Fig 3D,E).

### Cross-cohort expression-direction assessment and external sepsis validation of hub genes

**Fig 4:**
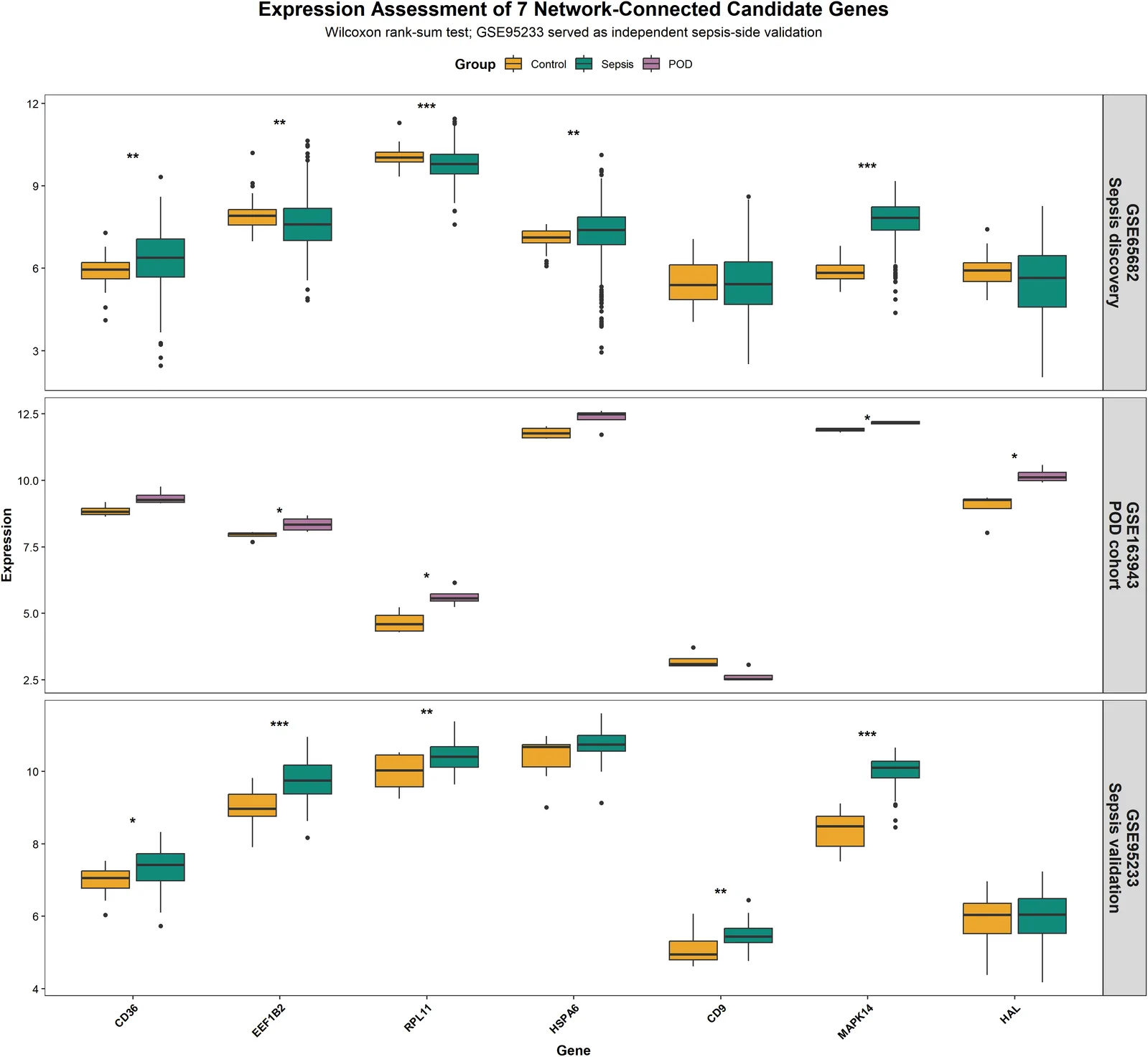
Cross-cohort expression-direction assessment and external sepsis validation of seven PPI-derived hub genes. Box plots show the normalized expression levels of CD36, EEF1B2, RPL11, HSPA6, CD9, MAPK14, and HAL in GSE65682 (sepsis discovery cohort, n = 802), GSE95233 (independent sepsis validation cohort, n = 79), and GSE163943 (POD cohort, n = 8). Disease and control groups were compared within each cohort using the Wilcoxon rank-sum test. Asterisks indicate nominal significance levels: *p < 0.05, **p < 0.01, and ***p < 0.001. Five of the seven genes were significantly differentially expressed in each sepsis cohort, and four were significant in the POD cohort. MAPK14 was significantly upregulated in all three cohorts, whereas the other genes showed cohort-dependent expression patterns. PPI, protein–protein interaction; POD, postoperative delirium; GSE, Gene Expression Omnibus Series.

To evaluate the cross-cohort expression patterns of the seven PPI-connected candidate genes, we compared their expression between disease and control groups in the sepsis discovery cohort (GSE65682), the POD cohort (GSE163943), and an independent sepsis validation cohort (GSE95233). Because no suitable independent POD transcriptomic cohort was available, external validation was limited to the sepsis dataset. Within each cohort, disease and control groups were compared using the Wilcoxon rank-sum test. Five of the seven genes were significantly differentially expressed in GSE65682, five were significant in GSE95233, and four were significant in GSE163943 (Fig 4). Directional consistency was not uniform across all genes. MAPK14 was significantly upregulated in all three cohorts, whereas CD36 and HSPA6 showed generally higher expression in the disease groups but did not reach nominal significance in every cohort. In contrast, EEF1B2 and RPL11 were downregulated in GSE65682 but upregulated in GSE95233 and GSE163943. These results identify MAPK14 as the candidate gene with the most consistent cross-cohort expression pattern.

### Diagnostic performance of hub genes

**Fig 5:**
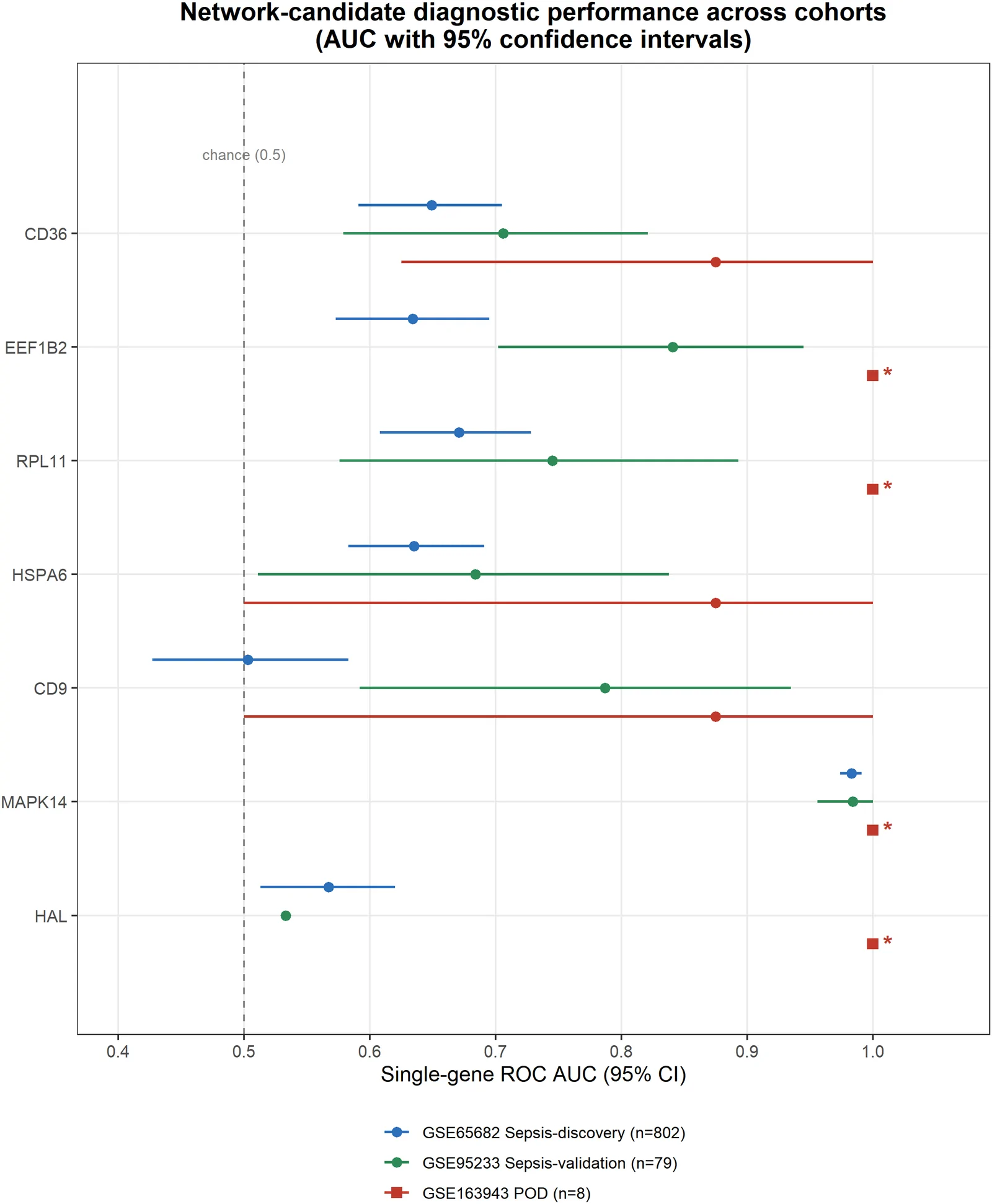
Single-gene ROC AUC with bootstrap 95% confidence intervals across cohorts. Points indicate AUCs for each of the seven PPI-derived hub genes, and horizontal bars indicate bootstrap 95% confidence intervals (CIs). Colors denote cohorts: blue, GSE65682 sepsis discovery cohort (n = 802); green, GSE95233 sepsis validation cohort (n = 79); and red, GSE163943 postoperative delirium (POD) cohort (n = 8). The dashed vertical line indicates chance-level discrimination (AUC = 0.5). MAPK14 showed consistently high AUCs in both sepsis cohorts, whereas the discriminatory performance of the remaining hub genes varied across cohorts. In the POD cohort, ROC estimates were unstable because of the very small sample size. Genes with complete class separation are marked by red squares with asterisks, and their degenerate CIs of 1.00–1.00 should not be interpreted as precise estimates of biomarker performance. POD ROC results were considered exploratory. ROC, receiver operating characteristic; AUC, area under the ROC curve; CI, confidence interval; PPI, protein–protein interaction; POD, postoperative delirium; GSE, Gene Expression Omnibus Series.

Single-gene ROC performance was evaluated using bootstrap 95% CIs and is presented as a forest plot (Fig 5). In the larger sepsis cohorts, the discriminatory performance of the seven PPI-derived hub genes varied substantially. In the discovery cohort (GSE65682, n = 802), MAPK14 showed the highest AUC (0.983, 95% CI: 0.974–0.991), followed by RPL11 (AUC = 0.671, 95% CI: 0.608–0.728) and CD36 (AUC = 0.649, 95% CI: 0.591–0.705), whereas the remaining genes showed relatively limited discriminatory performance. In the independent sepsis validation cohort (GSE95233), MAPK14 again showed the highest AUC (0.984, 95% CI: 0.956–1.000), followed by EEF1B2 (AUC = 0.841, 95% CI: 0.702–0.945), CD9 (AUC = 0.787, 95% CI: 0.592–0.935), and RPL11 (AUC = 0.745, 95% CI: 0.576–0.893). CD9 showed near-chance discriminatory performance in GSE65682 but a higher AUC in GSE95233, indicating limited cross-cohort diagnostic stability for this gene. MAPK14 showed consistently high discriminatory performance across both sepsis cohorts (AUC = 0.983 and 0.984).

In the underpowered POD cohort (n = 4 vs. 4), several genes reached apparent perfect AUCs; however, these estimates are likely inflated and statistically unstable because of complete class separation in a very small sample. The corresponding Wilcoxon P values were close to the minimum attainable value of approximately 0.03, and bootstrap CIs for perfectly separated genes were degenerate rather than informative. Therefore, diagnostic interpretation was based primarily on the larger sepsis cohorts, whereas the POD AUC results were treated as exploratory.

### SHAP-Based Interpretation of the Seven-Hub-Gene Model

**Fig 6:**
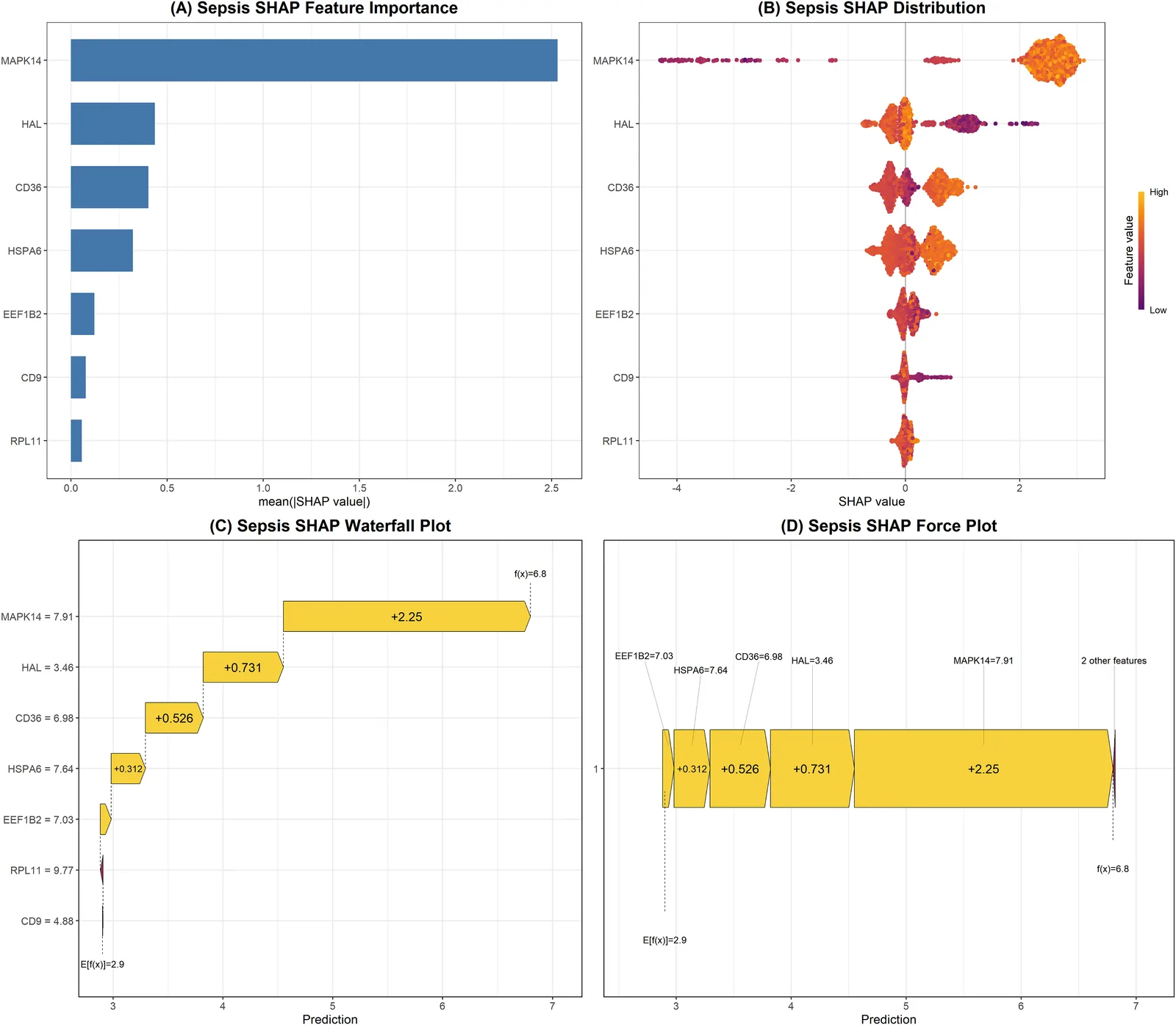
SHAP-based interpretation of the seven-hub-gene XGBoost classifier in the GSE65682 sepsis discovery cohort. (A) Bar plot showing the mean absolute SHAP values for the seven PPI-derived hub genes; MAPK14 had the highest feature contribution. (B) Beeswarm plot showing the distribution of SHAP values for each hub gene. Dot color represents the expression level of each gene, with positive SHAP values shifting predictions toward sepsis and negative values shifting predictions toward control. (C) Waterfall plot for a representative sample showing the additive contributions of individual hub genes to the model output. (D) Force plot illustrating the cumulative contributions of the hub genes from the baseline value to the final model output for the same representative sample. SHAP, SHapley Additive exPlanations; XGBoost, extreme gradient boosting; PPI, protein–protein interaction; GSE, Gene Expression Omnibus Series.

An XGBoost classifier was trained using the expression profiles of the seven PPI-derived hub genes in the sepsis discovery cohort GSE65682 (n = 802) to assess the relative contribution of each hub gene to sepsis classification. The POD cohort (4 controls and 4 POD cases) was not used for model training because its sample size was insufficient for reliable classifier fitting. SHAP analysis identified MAPK14 as the dominant contributor to model predictions, with the highest mean absolute SHAP value (mean |SHAP| = 2.531), followed by HAL (0.436), CD36 (0.402), and HSPA6 (0.322) (Fig 6A,B). The SHAP distribution plot showed that higher MAPK14, CD36, and HSPA6 expression tended to shift predictions toward the sepsis class, whereas lower HAL expression generally contributed positively to sepsis prediction (Fig 6B). Waterfall and force plots for a representative sample further illustrated the additive contributions of individual hub genes to the model output (Fig 6C,D). Overall, the SHAP-based ranking prioritized MAPK14 as the leading predictive feature among the seven PPI-derived hub genes in the sepsis discovery cohort. To assess potential overfitting, model calibration was additionally evaluated using repeated stratified five-fold cross-validation with Brier score and calibration-curve analyses; these results are provided in Supplementary S6 Fig.

### Identification of losartan as a mechanism-guided MAPK14-linked candidate

**Fig 7:**
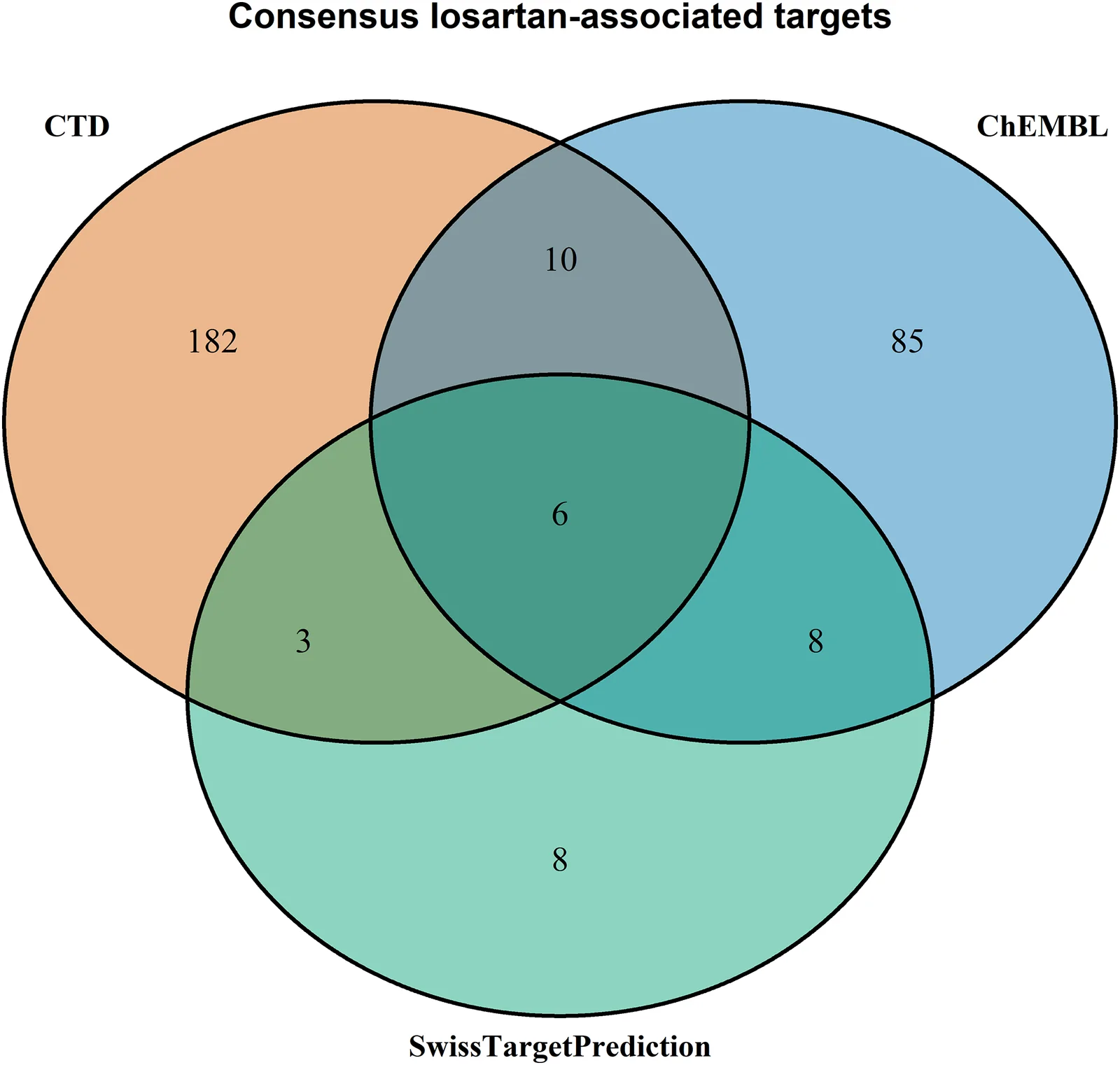
Consensus losartan-associated targets across CTD, ChEMBL, and SwissTargetPrediction. Venn diagram showing losartan-associated targets retrieved from CTD, ChEMBL, and SwissTargetPrediction. The intersection of the three databases yielded six consensus targets: ACE, AGTR1, AGTR2, MAPK1, MAPK14, and PTGS2. CTD, Comparative Toxicogenomics Database; ChEMBL, a bioactivity database maintained by the European Molecular Biology Laboratory–European Bioinformatics Institute.

After MAPK14/p38α was prioritized through the corrected transcriptomic, PPI, cross-cohort expression, and SHAP analyses, pharmacological candidates relevant to this target were considered. Losartan was selected for clinically informed, target-centered exploratory evaluation based on drug–gene evidence, prior literature, clinical availability, established human use, and the relevance of the renin–angiotensin system to inflammatory signaling. Curated CTD records further report that losartan decreases MAPK14 expression (PubMed identifiers [PMIDs]: 28091615 and 34590760). To assess whether the losartan–MAPK14 association was supported across multiple pharmacological resources, losartan-associated targets were retrieved from CTD, ChEMBL, and SwissTargetPrediction. The three-way intersection yielded six consensus losartan-associated targets, among which MAPK14 was the only gene overlapping with the seven network-connected candidate genes (Fig 7). These findings support MAPK14 as a plausible molecular link between losartan pharmacology and the shared sepsis–POD transcriptional signature, although this interpretation remains hypothesis-generating and requires experimental validation.

### Molecular docking of losartan with MAPK14/p38α

**Fig 8:**
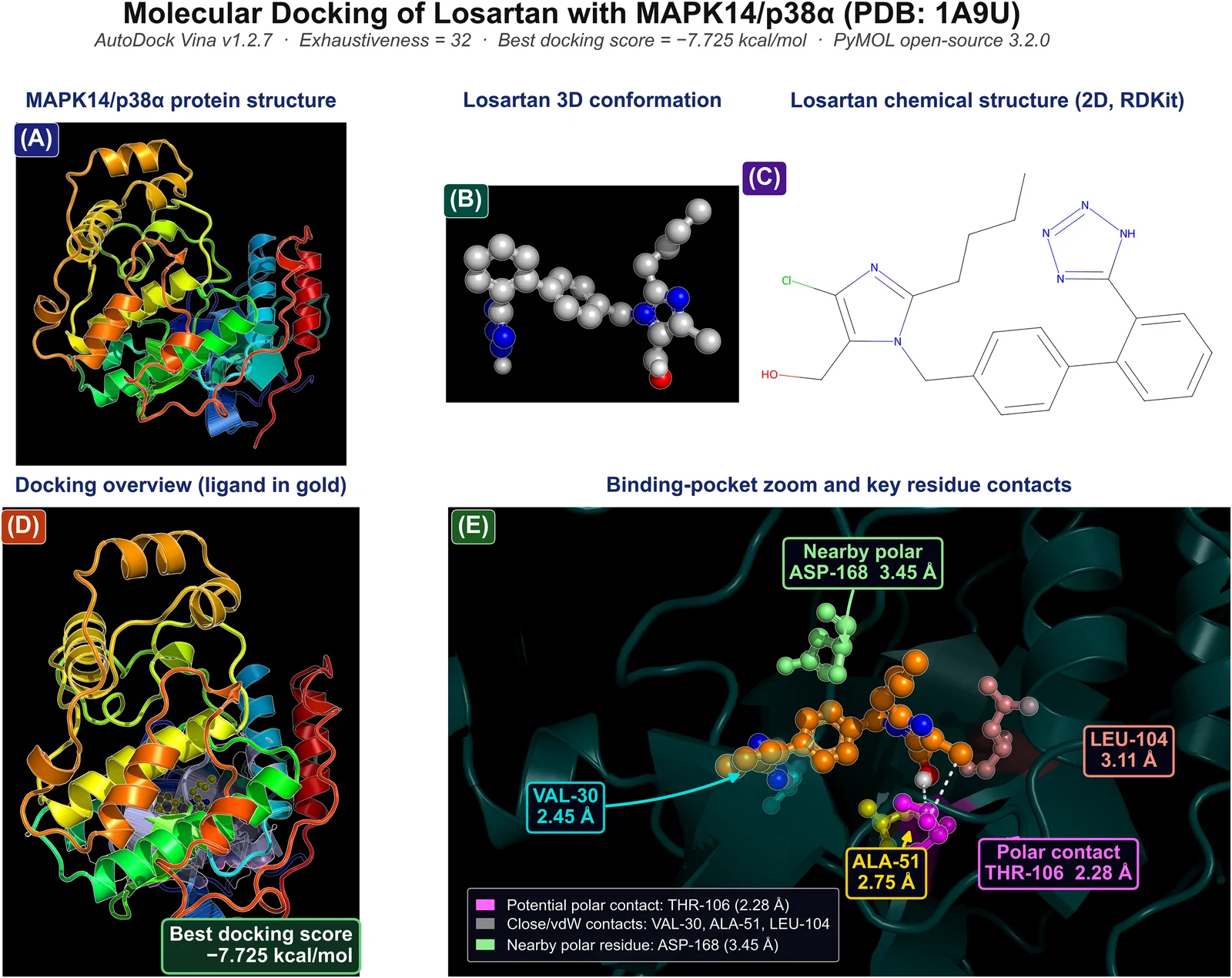
Molecular docking visualization of losartan with MAPK14/p38α. (A) Rainbow ribbon representation of the MAPK14/p38α crystal structure (PDB ID: 1A9U). (B) Three-dimensional conformation of losartan used for docking. (C) Two-dimensional chemical structure of losartan (PubChem CID: 3961). (D) Docking overview showing the best-ranked predicted losartan pose within the ATP-binding pocket of MAPK14/p38α. (E) ATP-pocket zoom of the best-ranked predicted pose, highlighting geometric proximity between losartan and nearby residues including ASP-168, ARG-173, and neighboring pocket residues. The best predicted docking score was ΔG = −7.725 kcal/mol. Docking was performed using AutoDock Vina v1.2.7 with exhaustiveness set to 32. PDB, Protein Data Bank; CID, compound identifier; ATP, adenosine triphosphate; ASP, aspartic acid; ARG, arginine; ΔG, predicted docking score/free-energy estimate.

**Fig 9:**
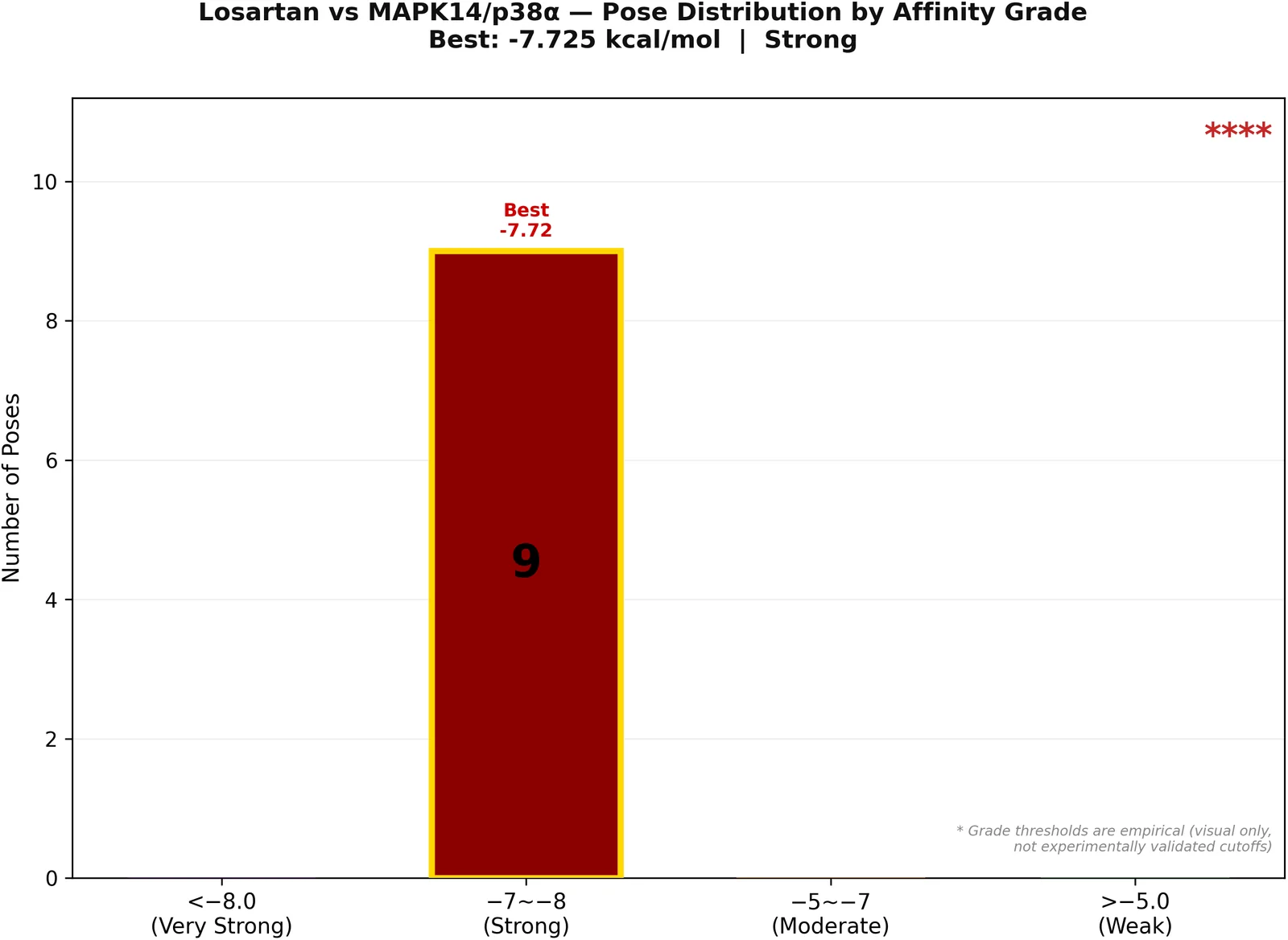
Predicted docking-score distribution of losartan-MAPK14/p38α docking poses. Bar chart showing the distribution of nine predicted docking poses according to predefined descriptive docking-score ranges. All nine poses fell within the favorable docking-score range, defined as −8.0 < ΔG ≤ −7.0 kcal/mol. The best-ranked pose showed a predicted docking score of ΔG = −7.725 kcal/mol. These empirical thresholds were used only for descriptive interpretation of docking scores and do not establish direct biochemical binding. ΔG, predicted docking score/free-energy estimate.

**Fig 10:**
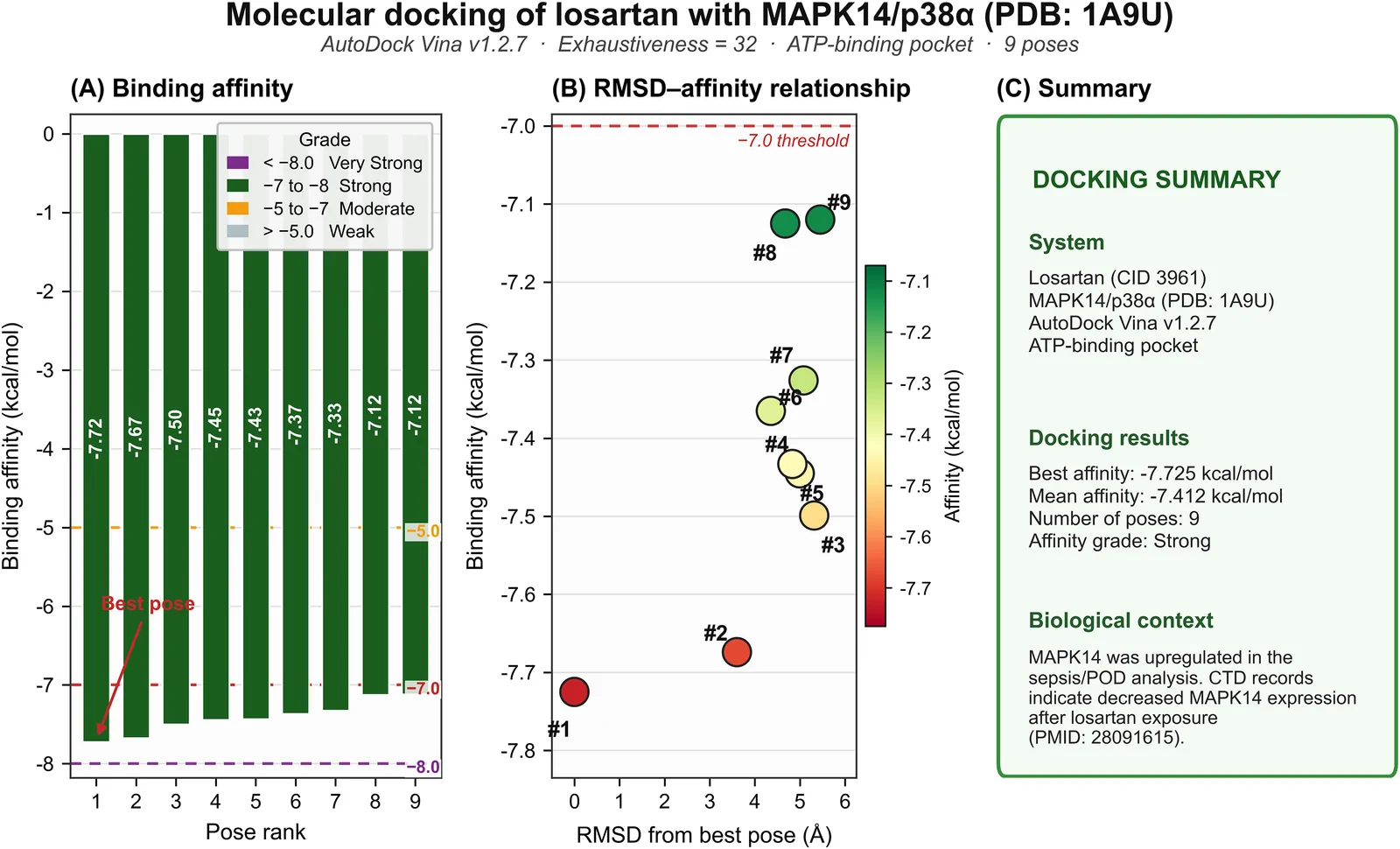
Docking score distribution and summary of losartan-MAPK14/p38α docking poses. (A) Bar chart showing the predicted docking scores of all nine docking poses (range: −7.120 to −7.725 kcal/mol). Dashed horizontal lines indicate predefined descriptive docking-score thresholds. (B) Scatter plot showing RMSD from the best-ranked pose versus predicted docking score for poses 1-9. (C) Summary panel showing docking parameters and descriptive results, including the best predicted docking score, mean docking score, number of poses, and overall descriptive score range. All nine poses fell within the favorable docking-score range, with a mean predicted score of −7.412 kcal/mol. RMSD, root-mean-square deviation.

Molecular docking of losartan (PubChem compound identifier [CID]: 3961) into the adenosine triphosphate (ATP)-binding pocket of MAPK14/p38α (PDB ID: 1A9U) using AutoDock Vina v1.2.7 generated nine predicted docking poses. The best-ranked pose was located within the ATP-binding pocket (Fig 8). Predicted docking scores ranged from −7.120 to −7.725 kcal/mol, with a mean score of −7.412 kcal/mol (Fig 9, Fig 10, Table 2). These results support structural plausibility for a losartan-MAPK14/p38α association, but they do not establish direct biochemical binding or functional inhibition.

**Table 2:** Predicted docking scores and key residue distances for nine losartan-MAPK14/p38α docking poses.

Pose	Docking score (kcal/mol)	RMSD_lb (Å)	RMSD_ub (Å)	ASP-168 (Å)	ARG-173 (Å)	Descriptive score range
1	−7.725	0.00	0.00	3.36	8.41	Favorable
2	−7.674	3.60	8.52	2.55	7.33	Favorable
3	−7.499	5.32	8.38	3.01	3.35	Favorable
4	−7.445	5.00	8.50	2.93	3.75	Favorable
5	−7.433	4.83	7.95	2.31	3.02	Favorable
6	−7.365	4.36	7.85	3.34	6.05	Favorable
7	−7.326	5.08	6.77	2.38	3.37	Favorable
8	−7.125	4.67	8.36	3.25	6.81	Favorable
9	−7.120	5.45	7.62	2.00	3.45	Favorable

Docking was performed using AutoDock Vina v1.2.7 with exhaustiveness set to 32 based on the MAPK14/p38α crystal structure from the Protein Data Bank (PDB ID: 1A9U). ΔG denotes the predicted docking score/free-energy estimate; RMSD_lb/RMSD_ub, lower-bound/upper-bound root-mean-square deviation from the best-ranked pose; ASP, aspartic acid; ARG, arginine; Å, angstrom. The ASP-168 and ARG-173 columns report the minimum heavy-atom distance between losartan and each residue. The favorable docking-score range (−8.0 < ΔG ≤ −7.0 kcal/mol) is used descriptively and does not establish direct biochemical binding.

Residue-distance analysis showed that ASP-168 was in close geometric proximity to losartan across all nine predicted poses, with a distance of 3.36 Å in the best-ranked pose and an overall range of 2.00–3.36 Å. ARG-173 showed pose-dependent proximity, with close heavy-atom contact in poses 3, 5, 7, and 9, but not in the best-ranked pose (8.41 Å). Thus, ASP-168 represented the most consistent nearby pocket residue, whereas ARG-173 involvement varied across predicted poses.

### Immune-Cell Composition and MAPK14 Expression

**Fig 11:**
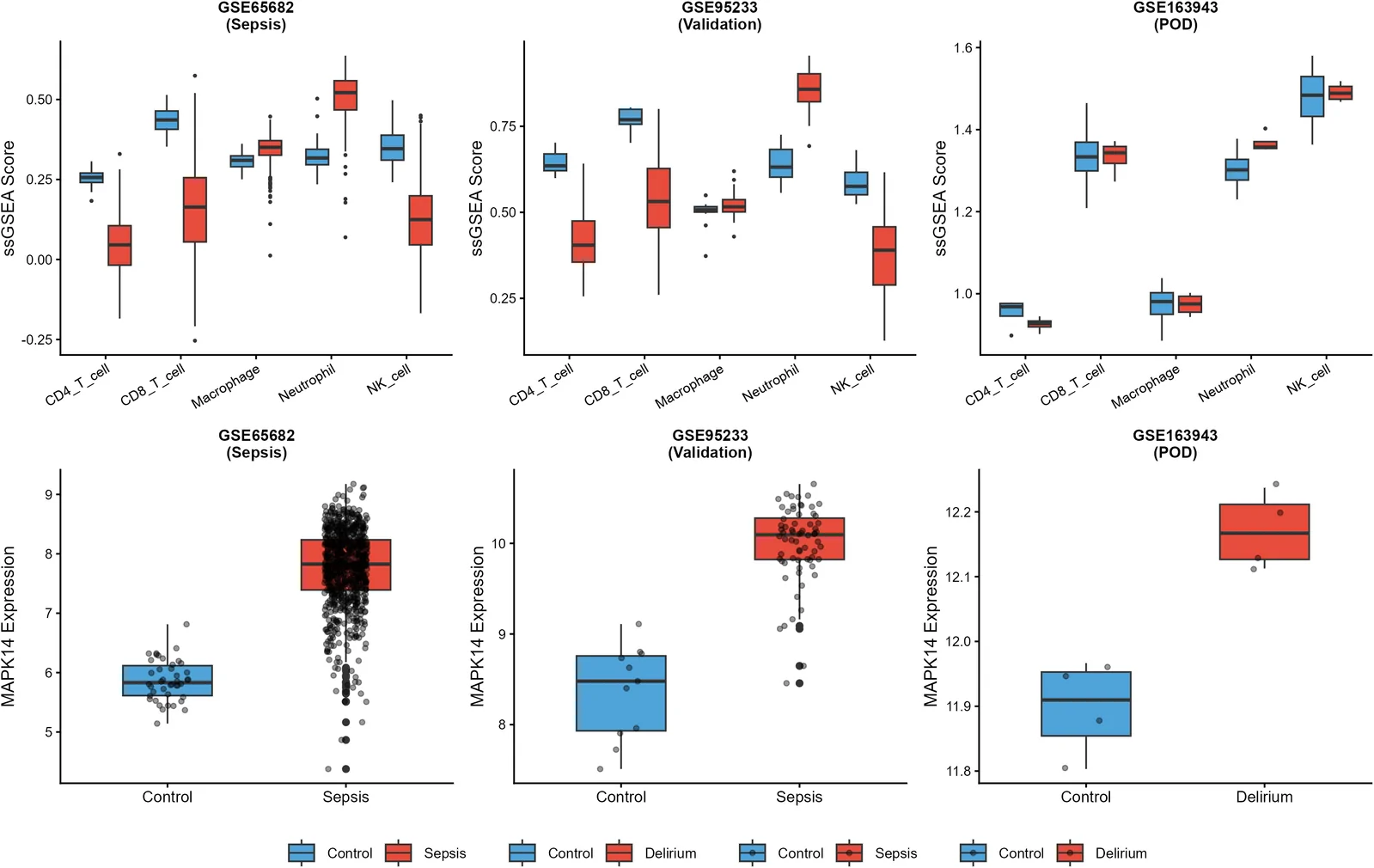
Immune-cell composition scores and MAPK14 expression across sepsis and postoperative delirium cohorts. Top row shows ssGSEA-derived immune-cell scores for five major immune cell types, including CD4 ⁺ T cells, CD8 ⁺ T cells, macrophages, neutrophils, and NK cells, across GSE65682, GSE95233, and GSE163943. Bottom row shows MAPK14 expression levels comparing disease and control groups in each cohort. Disease groups showed increased neutrophil ssGSEA scores and higher MAPK14 expression across the analyzed cohorts, with variable changes in other immune-cell subsets. ssGSEA, single-sample Gene Set Enrichment Analysis; POD, postoperative delirium; NK, natural killer. GSE, Gene Expression Omnibus Series; CD, cluster of differentiation.

To determine whether the shared inflammatory transcriptomic signature corresponded to innate immune-cell changes, particularly neutrophil-related alterations, we performed ssGSEA-based immune-cell composition analysis. Across the three cohorts, disease samples showed higher neutrophil scores and lower CD4 ⁺ T-cell and NK-cell scores than controls (Fig 11, upper panels). Macrophage scores were also increased in both sepsis cohorts, whereas this pattern was less evident in the POD cohort. Consistent with differential-expression analysis, MAPK14 was upregulated in disease samples across all three cohorts (Fig 11, lower panels).

**Fig 12:**
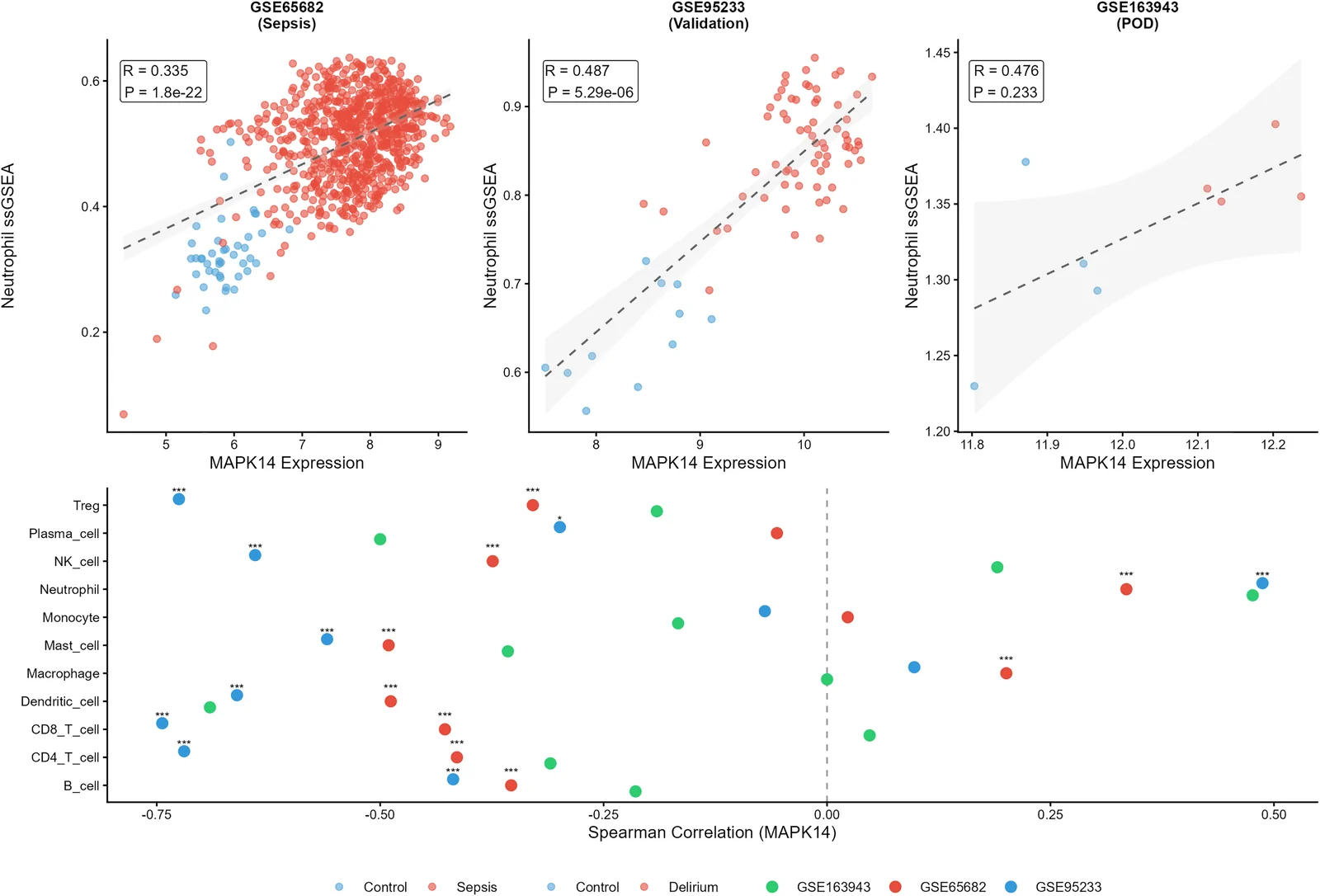
Correlation between MAPK14 expression and immune-cell ssGSEA signatures. Top row shows Pearson correlation analyses between MAPK14 expression and neutrophil ssGSEA scores in GSE65682, GSE95233, and GSE163943. Bottom panel shows Spearman correlations between MAPK14 expression and 11 immune-cell ssGSEA signatures across the three cohorts. Dot color indicates cohort, and asterisks indicate statistical significance after multiple-testing correction. MAPK14 showed consistent positive associations with neutrophil ssGSEA scores and negative associations with adaptive immune-cell signatures, including CD4 ⁺ T cells, CD8 ⁺ T cells, and B cells. ssGSEA, single-sample Gene Set Enrichment Analysis; FDR, false discovery rate; POD, postoperative delirium. GSE, Gene Expression Omnibus Series; CD, cluster of differentiation.

To further link MAPK14 to neutrophil-related immune alterations, we examined correlations between MAPK14 expression and ssGSEA immune-cell scores. MAPK14 expression was positively correlated with neutrophil scores in GSE65682 (Pearson R = 0.335, p = 1.8 × 10 ^⁻^ ^22^) and GSE95233 (R = 0.487, p = 5.29 × 10 ^⁻^ ^5^). A similar positive trend was observed in GSE163943 (R = 0.476, p = 0.233), although it did not reach statistical significance, likely reflecting the limited sample size. Spearman correlation analysis further showed that MAPK14 expression was negatively associated with adaptive immune-cell signatures, including CD4 ⁺ T cells, CD8 ⁺ T cells, and B cells. These findings suggest that MAPK14 upregulation is associated with a neutrophil-predominant innate immune signature, providing cellular-level context for the shared inflammatory signal observed in sepsis and POD (Fig 12).

### Hypothetical signaling model

**Fig 13:**
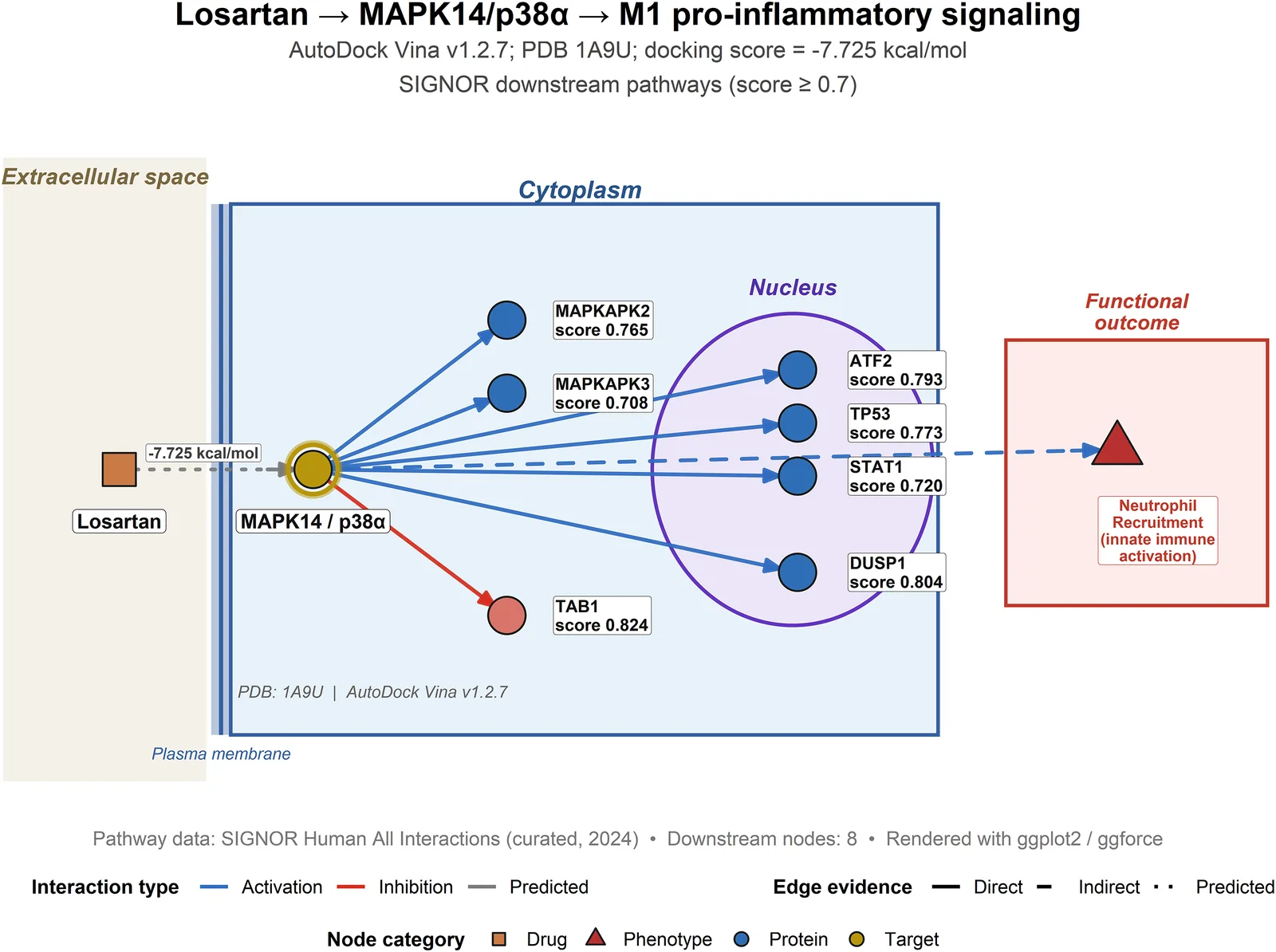
Hypothetical losartan-MAPK14/p38α signaling model linked to neutrophil-associated innate immune activation. Schematic diagram illustrating a putative drug-target-pathway axis derived from integrated computational analyses. Molecular docking suggested structurally plausible docking poses of losartan within the MAPK14/p38α ATP-binding pocket, but does not establish direct binding or functional inhibition. The downstream signaling network was curated from the SIGNOR database. High-confidence nodes included MAPKAPK2, MAPKAPK3, ATF2, TP53, STAT1, DUSP1, and TAB1, with SIGNOR confidence scores ≥0.70. The model suggests that losartan may be associated with MAPK14/p38α-related inflammatory signaling and neutrophil-associated innate immune activation in the shared inflammatory context of sepsis and postoperative delirium. Blue edges indicate activation, red edges indicate inhibition; solid, dashed, and dotted lines indicate direct, indirect, and predicted relationships, respectively. ATP, adenosine triphosphate; SIGNOR, SIGnaling Network Open Resource.

Based on the integrated computational evidence, we constructed a hypothetical signaling model linking losartan, MAPK14/p38α, and downstream inflammatory pathways. In this model, losartan is proposed as a candidate compound associated with MAPK14/p38α signaling, rather than as an experimentally confirmed MAPK14 inhibitor. The downstream network included MAPKAPK2, MAPKAPK3, STAT1, ATF2, TP53, DUSP1, and TAB1, all of which showed high-confidence regulatory connections in the SIGnaling Network Open Resource (SIGNOR) database. The corresponding SIGNOR confidence scores ranged from 0.708 to 0.824. This putative axis may be related to neutrophil-associated innate immune activation in the shared sepsis–POD inflammatory context (Fig 13). As this model was derived from transcriptomic integration, drug-target database evidence, molecular docking, and pathway-network analysis, it should be interpreted as hypothesis-generating and requires experimental validation.

## Discussion

Sepsis and postoperative delirium (POD) are independently associated with substantial morbidity and long-term neurocognitive impairment [14,15], and the occurrence of POD in patients with sepsis may further aggravate clinical deterioration; yet their shared molecular relationship has remained poorly understood. A critical conceptual point motivating this study is that the two conditions arise from mechanistically distinct triggers—microbial invasion and systemic infection in sepsis, versus surgical trauma and anaesthesia-related stress in POD—yet both culminate in systemic immune dysregulation, neuroinflammation, and blood–brain barrier disruption [16,17]. The transcriptomic overlap identified here therefore does not simply reflect clinical co-occurrence; rather, it suggests that diverse injurious stimuli converge on a shared set of downstream immune-inflammatory effector pathways, irrespective of the initiating aetiology. This mechanistic commonality is of particular clinical relevance for patients who develop sepsis in the perioperative setting, where both pathological axes are simultaneously active and may synergistically amplify neurocognitive deterioration. Previous bioinformatics studies have examined the transcriptomic landscape of sepsis [18,19] or POD-related cognitive dysfunction [20] in isolation; to our knowledge, however, few studies have applied an integrated multi-cohort transcriptomic framework to systematically identify shared hub genes at the PPI network level across both diseases simultaneously, nor coupled this analysis with machine learning-based feature prioritization and multi-database pharmacological cross-checking to nominate a mechanism-guided, drug-linked candidate hub. In the present study, we identified seven hub genes at this transcriptomic intersection through four-algorithm PPI centrality consensus and validated their expression across three independent cohorts, with MAPK14/p38α emerging as the leading candidate hub by network centrality and SHAP importance and as the sole hub gene overlapping with losartan’s multi-database target profile.

Functional enrichment of the 22 directionally concordant shared DEGs indicated a heterogeneous signature involving oxidative stress, hemostasis, granule-associated processes, and tissue remodeling, many of which are closely linked to immune-inflammatory regulation. This pattern is consistent with the broader framework of sepsis-associated immune dysregulation, in which excessive inflammatory activation may coexist with or be followed by sustained immunosuppression [21,22]. This framework may also be relevant to the perioperative inflammatory state associated with POD, in which an initial pro-inflammatory response may coexist with or be followed by compensatory immune suppression. In critically ill patients, this immunosuppressive phase is characterized by T-lymphocyte dysfunction, monocyte deactivation, and reduced HLA-DR expression and has been associated with secondary infection and adverse outcomes [23,24]. Reduced HLA-DR expression on circulating monocytes is a recognized marker of sepsis-associated immunoparalysis, and immune-restorative strategies involving GM-CSF or IL-7 have been proposed to counteract this suppression [25,26]. Although our enrichment results did not indicate a specific MHC class II or T-cell differentiation signature, the identified stress-response and granule-associated processes are consistent with broader immune-inflammatory dysregulation that may also contribute to perioperative neuroinflammation and POD.

Among the network-connected candidates, MAPK14 showed the most consistent upregulation across the sepsis discovery cohort, the independent sepsis cohort, and the POD cohort. Together with the enrichment of granule-associated processes and the positive association between MAPK14 expression and neutrophil ssGSEA scores, this finding suggests a potential link between MAPK14 and neutrophil-associated innate immune activation in the shared sepsis–POD inflammatory context. However, these associations do not establish a causal role for MAPK14 in neutrophil activation. Molecular docking further identified structurally plausible poses of losartan within the MAPK14/p38α ATP-binding pocket, with predicted docking scores ranging from −7.120 to −7.725 kcal/mol. This observation is compatible with previous experimental evidence that losartan attenuated p38 MAPK phosphorylation in a murine hepatic ischemia–reperfusion model [27], but does not demonstrate direct losartan–MAPK14 binding or functional MAPK14 inhibition.

Among the seven network-connected candidate genes, MAPK14/p38α warrants particular mechanistic attention because it showed the most consistent cross-cohort upregulation and the highest SHAP contribution. p38α is a serine/threonine kinase that integrates inflammatory and stress-related signals, including TLR/MyD88 signaling, reactive oxygen species, and damage-associated molecular patterns, through upstream kinases such as MKK3 and MKK6 [28]. Through its canonical downstream substrate MAPKAPK2, p38α can stabilize AU-rich element-containing transcripts encoding inflammatory mediators such as TNF-α, IL-6, and IL-1β, thereby sustaining inflammatory signaling [29,30]. p38α also regulates ATF2- and p53-related stress and apoptotic pathways [31,32], providing a plausible mechanistic link between systemic inflammatory stress and downstream cellular injury. Experimental studies have further implicated p38 MAPK signaling in microglial activation and neuroinflammatory responses, although the specific disease models and interventions have varied [33–36].

Single-gene ROC analysis was used as an exploratory assessment of individual candidate-gene discrimination rather than as a definitive diagnostic model. Discriminatory performance varied among the seven network-connected candidate genes. MAPK14 showed consistently high AUCs in both sepsis cohorts, whereas POD-specific estimates were statistically unstable because of the 4 vs. 4 sample size and complete class separation for several genes. MAPK14 also had the highest SHAP contribution, which, together with its consistent cross-cohort upregulation, supported its prioritization as the candidate shared molecular target. After MAPK14 had been prioritized, MAPK14-associated pharmacological candidates were considered, and losartan was selected for exploratory evaluation based on drug–gene evidence, prior literature, clinical availability, established human use, and mechanistic relevance. Subsequent cross-checking across CTD, ChEMBL, and SwissTargetPrediction yielded six consensus losartan-associated targets, among which MAPK14 was the only gene overlapping with the seven network-connected candidates. Curated database records and experimental studies further link losartan or angiotensin II type 1 receptor blockade to modulation of p38 MAPK-related expression and signaling, although these findings do not establish direct losartan–MAPK14 binding or selective inhibition of p38α [37–40]. Preclinical studies have reported that losartan attenuates neuroinflammatory, oxidative, and behavioral abnormalities in models of systemic inflammation and neurological injury [41,42]. However, observational evidence regarding perioperative renin–angiotensin system inhibitor exposure and delirium remains heterogeneous. Preoperative exposure was not associated with reduced delirium in a large postoperative critical-care cohort, although postoperative administration was associated with lower odds of delirium in a post hoc analysis; conversely, an inverse association between preoperative exposure and POD was reported in a smaller retrospective cohort restricted to patients with pulmonary arterial hypertension [43,44]. These observations provide preliminary clinical context but do not demonstrate that losartan prevents or treats sepsis-associated POD or that it is superior to other MAPK14-associated candidates.

Molecular docking was used solely to assess the structural plausibility of a losartan–MAPK14/p38α association. The nine predicted poses yielded docking scores ranging from −7.120 to −7.725 kcal/mol. ASP-168 was consistently located in close geometric proximity to losartan across all poses (2.00–3.36 Å), whereas the proximity of ARG-173 varied among poses. ASP-168 is located within the conserved DFG motif adjacent to the kinase active-site region [45]. However, geometric proximity in a docking model does not establish a specific molecular interaction or functional relevance. The predicted poses should therefore be interpreted as indicating that losartan can be computationally accommodated within the MAPK14/p38α ATP-binding pocket, rather than as evidence of direct binding or kinase inhibition. Confirmation will require biophysical binding assays and cellular or biochemical kinase-activity measurements.

Based on the integrated computational evidence, we constructed a hypothetical signaling model (Fig 13) in which losartan may influence MAPK14/p38α-associated inflammatory signaling indirectly through its established AT1R-blocking activity. Curated pathway relationships further connect MAPK14/p38α with downstream signaling components, including MAPKAPK2, MAPKAPK3, STAT1, ATF2, TP53, DUSP1, and TAB1. This putative network may be associated with neutrophil-related innate immune activation in the shared sepsis–POD inflammatory context. However, neither a direct losartan–MAPK14 interaction nor the proposed downstream signaling sequence was experimentally established in the present study. The model should therefore be regarded as a hypothesis-generating framework for future biochemical, cellular, and in vivo validation.

Several strengths and limitations should be considered. First, this study integrated three distinct GEO datasets spanning different clinical phenotypes and microarray platforms, allowing cross-cohort evaluation of candidate-gene expression. Second, candidate-gene prioritization did not depend on a single analytical step or significance threshold; instead, it combined RUV2-style-adjusted differential-expression analysis, identification of directionally concordant shared genes, threshold-independent RRHO-like concordance analysis, PPI network assessment, cross-cohort expression evaluation, and SHAP-based feature interpretation. Third, the pharmacological analysis followed a target-first framework. MAPK14 was prioritized before MAPK14-associated pharmacological candidates were considered, after which losartan was selected on the basis of available drug–gene evidence, prior literature, clinical availability, established human use, and mechanistic relevance. The losartan–MAPK14 association was subsequently cross-checked using CTD, ChEMBL, and SwissTargetPrediction and further evaluated through molecular docking. Together, these complementary analyses reduced dependence on any single dataset, statistical threshold, analytical method, or pharmacological resource.

Several limitations remain. First, the transcriptomic analyses were retrospective and cross-sectional and therefore cannot establish causal relationships between candidate-gene expression and disease pathophysiology. Second, the POD cohort included only four cases and four controls and was severely underpowered for genome-wide differential-expression analysis; consequently, the POD-specific differential-expression, single-gene expression, and ROC estimates should be regarded as exploratory. Third, although the RUV2-style adjustment [12,13] reduced test-statistic inflation in GSE65682 from λGC = 41.49 to λGC = 6.09, the residual λGC remained far above the expected range of 1.0–1.1, indicating severe remaining confounding that may reflect unmeasured clinical or technical heterogeneity. Accordingly, the RUV2-adjusted differential-expression results should be interpreted cautiously, and we emphasized directionally concordant genes, pathway-level findings, multistep candidate prioritization, and cross-cohort expression patterns rather than relying on isolated DEG calls. Fourth, molecular docking provides only an in silico assessment of structural plausibility and cannot establish direct binding or functional MAPK14 inhibition. The use of an SB203580-bound MAPK14 structure may also introduce conformational bias, and the predicted poses require validation through biophysical binding and biochemical or cellular kinase-activity assays. Fifth, although MAPK14-associated pharmacological candidates were considered before losartan was selected using clinically informed and literature-supported criteria, this approach did not comprehensively rank all possible MAPK14-associated compounds and therefore cannot establish losartan as the optimal or highest-ranked candidate. Moreover, direct evidence supporting a losartan–MAPK14 association in human cells or clinical settings remains limited. Finally, the bulk-transcriptomic design and ssGSEA-based immune-signature analysis cannot distinguish cell-intrinsic expression changes from shifts in cellular composition or attribute the observed signals to specific immune-cell populations; single-cell and spatial transcriptomic studies will therefore be required for cell-type-specific validation.

## Conclusion

In conclusion, this study identified MAPK14/p38α as a candidate shared molecular target between sepsis and POD through integrated transcriptomic, protein-interaction network, and machine-learning analyses. Losartan showed a hypothesis-generating association with MAPK14-related inflammatory signaling; however, the current evidence remains computational and does not establish direct binding, functional inhibition, or therapeutic efficacy. Further experimental and prospective clinical studies are required to validate the biological significance and therapeutic relevance of this finding.

## Supporting information

S1 Fig Sensitivity analysis of RUV2-style adjustment parameters in GSE65682. Genomic inflation factors and numbers of differentially expressed genes were compared across empirical control-gene proportions of 5%, 10%, and 20% and k values from 1 to 10. The final model used the 10% lowest-variance genes as empirical controls and k = 4 unwanted factors.
(TIFF)

S2 Fig Quantile–quantile plots before and after RUV2-style adjustment in GSE65682. The unadjusted model showed marked test-statistic inflation with λGC = 41.49. After RUV2-style adjustment using the 10% lowest-variance genes and k = 4, λGC decreased to 6.09, although substantial residual inflation remained.
(TIFF)

S3 Fig Quantile–quantile plots for the sepsis discovery and validation cohorts. Quantile–quantile plots are shown for the RUV2-adjusted GSE65682 discovery cohort and the independent GSE95233 validation cohort. The corresponding genomic inflation factors were λGC = 6.09 and λGC = 6.25, respectively.
(TIFF)

S4 Fig Quantile–quantile plot of the RUV2-adjusted differential-expression analysis in GSE65682. Observed and expected −log10(P) values are shown after adjustment using the 10% lowest-variance genes and four unwanted factors. The genomic inflation factor remained elevated at λGC = 6.0854.
(TIFF)

S5 Fig Quantile–quantile plot of the differential-expression analysis in GSE163943. Observed and expected −log10(P) values are shown for the postoperative delirium cohort. The estimated genomic inflation factor was λGC = 1.349.
(TIFF)

S6 Fig Cross-validated calibration of the hub gene XGBoost model in GSE65682. Model calibration was assessed using repeated stratified five-fold cross-validation with 20 repeats. The analysis yielded a mean Brier score of 0.007 ± 0.001 and a mean area under the receiver operating characteristic curve of 0.998 ± 0.001.
(TIFF)

S1 Table Summary of differential-expression results and genomic inflation in the initial analyses. The table presents the numbers of tested genes, log2 fold-change thresholds, differentially expressed gene counts, significance criteria, and genomic inflation factors for the initial GSE65682 sepsis and GSE163943 postoperative delirium analyses.
(CSV)

S2 Table Statistical power assessment of the included transcriptomic cohorts. The table summarizes sample sizes, Bonferroni-adjusted significance thresholds, minimum detectable effect sizes, and estimated statistical power for GSE65682, GSE95233, and GSE163943.
(CSV)
